# Error in Visual Abstract

**DOI:** 10.1001/jamanetworkopen.2023.4174

**Published:** 2023-02-24

In the Original Investigation titled “Assessment of Quality of Life Among Patients With Recurrent *Clostridioides difficile* Infection Treated with Investigational Oral Microbiome Therapeutic SER-109: Secondary Analysis of a Randomized Clinical Trial”^1^ published January 30, 2023, there was a mistake in the Visual Abstract. In the Findings section, the labels for the placebo and SER-109 were inadvertently transposed. This article has been corrected.^1^
